# Exploring Rehabilitation Patients’ Perspectives on What Matters for the Adoption of Home-Based Rehabilitation Technology: Q-Methodology Study

**DOI:** 10.2196/71515

**Published:** 2025-07-09

**Authors:** Karlijn E te Boekhorst, Sanne J Kuipers, Gerard M Ribbers, Jane M Cramm

**Affiliations:** 1 Department of Socio-Medical Sciences, Erasmus School of Health Policy & Management, Erasmus University Rotterdam, Burgemeester Oudlaan 50, Rotterdam, 3062 PA, The Netherlands, 31 10 408 8555; 2 Department of Rehabilitation Medicine, Erasmus MC, Rotterdam, The Netherlands

**Keywords:** home-setting, rehabilitation technology, Q-methodology, technology acceptance, acquired brain injury, patient views, home-based rehabilitation technology

## Abstract

**Background:**

Rehabilitation technologies can support recovery and rehabilitation outside clinical settings. However, their adoption remains challenging. Factors such as ease of use, perceived benefits, and social influence play a role, but little is known about how rehabilitation patients perceive their relative importance.

**Objective:**

This study aimed to systematically explore the viewpoints of rehabilitation patients regarding the adoption of home-based rehabilitation technology.

**Methods:**

Between May and September 2024, this study examined the viewpoints of rehabilitation patients with acquired brain injury regarding the adoption of home-based rehabilitation technology using Q-methodology. A purposive sample of 21 participants ranked 34 opinion statements based on perceived importance and explained their choices during follow-up interviews. By-person factor analysis identified common patterns in how participants ranked the statements. These patterns, referred to as factors or viewpoints, were further interpreted using qualitative interview data.

**Results:**

Three viewpoints were identified, each highlighting different factors important for adopting home-based rehabilitation technology: (1) technology supporting rapid recovery, (2) technology supporting independence and self-control, and (3) technology as a supporting partner. Participants consistently emphasized the importance of regaining independence, receiving feedback during exercises, simple and easy-to-use designs, and approval from therapists, while positive reports in mainstream media, support from friends, and reducing travel to rehabilitation centers were considered less important.

**Conclusions:**

The findings suggest that rehabilitation patients with acquired brain injury prioritize different factors when adopting home-based rehabilitation technology. While some factors are commonly valued, the diversity in patient viewpoints underscores the need for tailored, user-centered approaches in the design and implementation of these technologies. A one-size-fits-all approach would likely be ineffective in meeting their varying needs.

## Introduction

Rehabilitation technology is a rapidly growing field in both research and clinical practice [1,2]. These technologies encompass tools and devices designed to support individuals in improving or regaining physical, cognitive, psychological, or social functioning following injury or illness [3]. They offer several benefits, such as enabling home-based training [4,5], reducing the workload of health care professionals [6], and accelerating recovery through increased therapy intensity and patient engagement [4,5,7]. However, their adoption in home settings remains challenging, with abandonment rates reaching up to 80% [8,9].

The reasons for abandonment are multifaceted and often stem from a mismatch between users’ needs and preferences and the available technology [10-14]. The technology may not always address users’ needs, and as those needs evolve, initially suitable technologies may become inadequate [13]. This highlights the necessity of user-centered approaches in the development of home-based rehabilitation technology [10,11]. Incorporating users’ perspectives throughout the development process is considered essential for improving usability and adoption rates.

The unified theory of acceptance and use of technology (UTAUT) [15] is a framework that can provide insights into users’ perspectives regarding technology adoption. UTAUT synthesizes elements from 8 established models, including the theory of reasoned action [16], the theory of planned behavior [17], and the technology acceptance model [18]. While the 8 models individually explain 17%‐42% of the variance in technology acceptance, UTAUT accounts for up to 69% [15], making it a robust framework for understanding technology adoption behavior and informing design and implementation practices.

According to UTAUT, 3 main factors determine the intention to adopt technology: performance expectancy (the perceived benefits), effort expectancy (the ease of use), and social influence (the belief that significant others support its use). This intention, combined with facilitating conditions such as available resources, ultimately determines adoption behavior [15]. These factors may also be influenced by individual characteristics, such as age, sex, education level, and disease severity [15,19-23].

The relative importance of these adoption factors may differ between rehabilitation patients, as each follows a personalized treatment plan based on their specific impairments and goals [24]. Exploring this variation and identifying commonalities are therefore crucial for the effective design and implementation of home-based rehabilitation technology. This study aims to systematically explore the viewpoints of rehabilitation patients using the UTAUT framework and Q-methodology. Specifically, the focus is on rehabilitation patients with acquired brain injury (ABI). With an aging population, the number of patients with ABI requiring rehabilitation is expected to rise, while resources remain limited [25,26]. Home-based rehabilitation technology has the potential to counterbalance this trend, but only if it is successfully and widely adopted. By focusing on the viewpoints of rehabilitation patients with ABI, this study seeks to provide insights that can guide the development and implementation of home-based rehabilitation technology.

## Methods

### Study Design

A Q-methodology study (Q-study) was conducted. This is a mixed methods approach that enables the systematic examination of experiences and perspectives on a specific topic within a particular population. Participants rank statements on a sorting grid based on their perceived importance or level of agreement, followed by interviews in which they elaborate on their reasoning. The ranked statements are analyzed using by-person factor analysis to identify shared viewpoints, which are then interpreted alongside qualitative interview data to construct a narrative for each viewpoint [27].

### Participants and Setting

The study was conducted at Rijndam, a rehabilitation center in Rotterdam, the Netherlands. Eligible participants were aged 18 years or older, diagnosed with ABI, had completed or were still undergoing inpatient or outpatient rehabilitation at Rijndam, and were proficient in Dutch. Prior experience with home-based rehabilitation technology was not required.

Health care professionals at the center informed patients about the study. Interested patients could either schedule an appointment directly or give permission for their health care provider to share their contact details with the first author (KEB), who then contacted them to schedule an appointment. Of the 22 patients who expressed interest, 1 was excluded due to a visual impairment. The final sample included 21 participants.

Participants were purposively selected to ensure maximum diversity of perspectives relevant to the study context. Theoretical saturation was monitored throughout recruitment. No substantially new opinions or opinion statements emerged in the final 3 interviews, indicating that the final sample was sufficient to capture the range of shared viewpoints present in this setting [28].

### Statement Development

To explore the full range of possible views on the topic, the statements in a Q-study need to encompass the subject comprehensively [27]. In this study, the statements were developed without a strict definition of specific rehabilitation technology to allow for authentic responses based on participants’ own interpretations. However, the scope was limited to technologies used for physical rehabilitation.

A structured approach was used to develop the statements. First, we conducted a review of peer-reviewed literature [10,19,20,29-36] on the acceptance and adoption of rehabilitation technologies, which resulted in 156 (overlapping) verbal statements. These were then organized according to the factors outlined in the UTAUT framework: performance expectancy, effort expectancy, social influence, facilitating conditions, and behavioral intention. Subsequently, the authors KEB, JMC, and SJK reviewed and refined the set of statements, reducing it to 34. Given that patients with ABI often experience cognitive and language impairments [37] and that too many statements could be overwhelming [27], extra care was taken to keep the statements concise while still covering the most important aspects. To ensure the language was accessible, the Dutch “Is het B1?” tool was used to check for B1-level phrasing. The statements were pilot-tested with 2 rehabilitation patients, who were invited to provide feedback on clarity and whether anything important was missing. Neither suggested any changes. Similarly, participants in the main study were asked whether any key points had been overlooked or if anything was unclear. No issues were raised. Table 1 presents the final set of statements.

**Table 1.** Final set of statements and idealized Q-sort for the factors (viewpoints).

Statement		Factor or viewpoint		
		^1Aa^	^2Bb^	^3Cc^
Performance expectancy				
	1. A faster recovery process	4^d^	1^d^	3^d^
	2. Reaching rehabilitation goals more quickly	4	0^d^	3
	3. Control over my own recovery process	0	4^d^	2
	4. The convenience of recovering at home	3	2	0^d^
	5. Flexibility to do exercises whenever I want	−1	4^d^	0
	6. Avoiding travel to the rehabilitation center	−2	−2	−1
	7. Being less dependent on others	1	1	2
Effort expectancy				
	8. Technology that is simple to use	0	0	1
	9. Technology that is quick and easy to understand	1^e^	0	−1
	10. Technology that is ready for immediate use	2	1	−1
	11. Being able to understand the technology without explanation	0	−1	−2
	12. Being able to independently use the technology	1	3^e^	1
	13. Setting up the technology within 5 minutes	2^d^	−1^e^	−2^e^
Social influence				
	14. Approval from my doctor or therapist	1	2	2
	15. Support from family	2^e^	−1	1
	16. Support from friends	−2	−2	−3
	17. Recommendations from peers	−2	−3	1^d^
	18. Positive experiences shared on social media	−4	−4	−4
	19. Positive coverage on television	−4	−4	−2
Facilitating conditions				
	20. Maintaining contact with peers	−3	−3	0^d^
	21. Progress monitored by a physician or therapist	3	2	4^e^
	22. Insight into my own progress	0^d^	3^e^	4^e^
	23. Direct feedback during exercises	3	1^e^	3
	24. The ability to compare my progress with peers	−1^d^	−2^d^	1^d^
	25. Rewards for performing exercises well	−2	−2	0^d^
	26. Technology with different levels of difficulty	1	−1^d^	2
	27. Exercises presented in the form of games	−3^d^	0	0
	28. Availability of support for technical issues	0	1^d^	−1
	29. Cost reimbursement for the technology	0	0	−2^e^
	30. Guarantee of my safety	2	3	0^d^
	31. Guarantee of my privacy	−3^e^	2^e^	−4^e^
	32. Limited space required for the technology	−1	−1	−3^d^
Intention to use				
	33. Technology as a supplement to treatment at the rehabilitation center	−1	0	−1
	34. Technology as a partial replacement for treatment at the rehabilitation center	−1^d^	−3	−3

a Technology supporting rapid recovery.

b Technology supporting independence and self-control.

c Technology as a supporting partner.

d *P*<.01.

e *P*<.05

### Data Collection

From May to September 2024, the first author (KEB) conducted individual Q-study interviews in person, in Dutch, lasting an average of 41 (SD 13) minutes. Interviews took place either at participants’ homes or at the rehabilitation center, depending on their preference. With consent, interviews were audio-recorded to ensure accuracy and for reference purposes.

**Figure 1.**
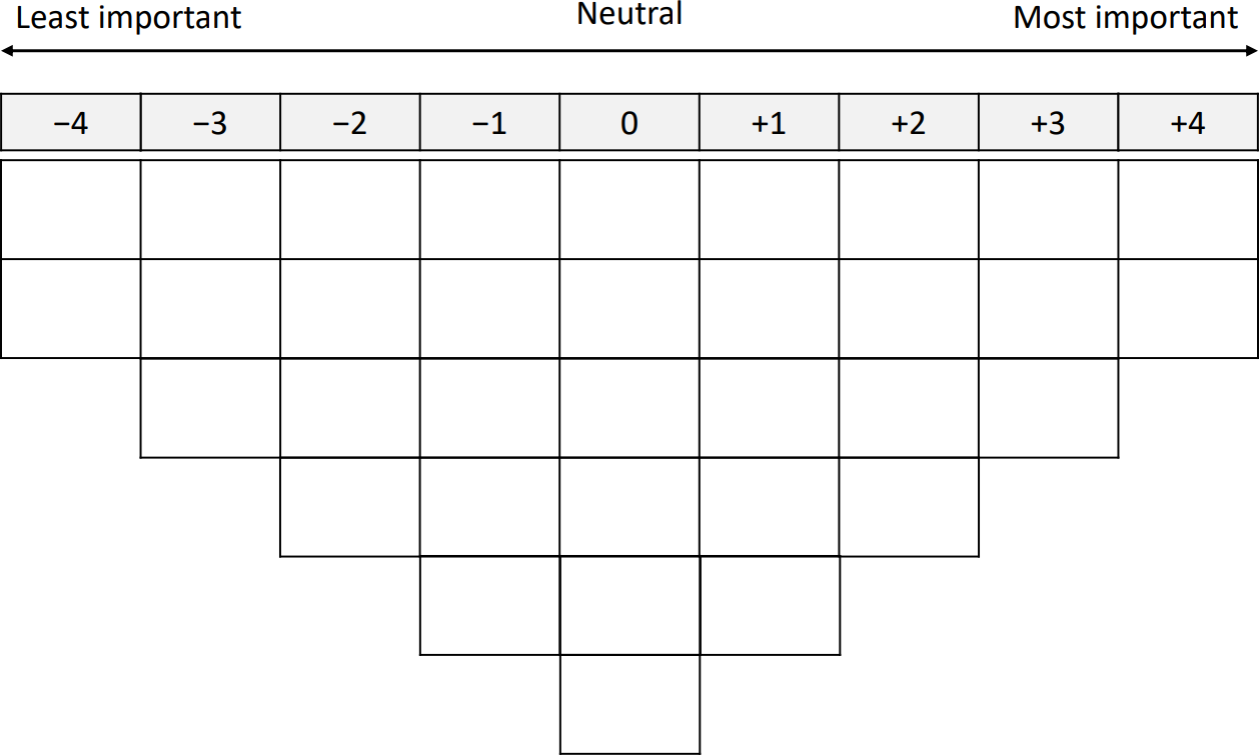
The sorting grid.

During the interview, participants were asked to sort the 34 statements into 3 initial piles: “important,” “neutral,” and “unimportant.” Once sorted, the statements were ranked on a sorting grid with a forced-choice frequency distribution ranging from −4=least important to +4=most important (Figure 1). Participants began by selecting the 2 most important statements from the “important” pile and placing them in the +4 column. They then selected 3 slightly less important statements for the +3 column. This process continued until all statements from the “important” pile had been ranked. The same procedure was repeated for the “unimportant” pile, beginning with the −4 column and moving up. Finally, the “neutral” statements were placed in the remaining columns. This process resulted in a fully ranked set of statements, commonly referred to as a Q-sort [27].

After completing the ranking, participants were asked to explain their choices, with particular attention to the statements placed in the extreme columns (+4 and −4). These explanations provided qualitative data that helped contextualize the rankings. All participant comments during the ranking process and explanations were transcribed verbatim for analysis.

### Data Analysis

The rankings of the 21 participants were analyzed using by-person factor analysis to identify shared viewpoints on the acceptance of rehabilitation technology. The analysis was conducted using KADE software (version 1.3.1) [38]. Data clusters were identified through centroid factor analysis and varimax rotation, with each cluster representing a potential factor, which in turn represents a potential shared viewpoint among participants. Potential factors were evaluated using 3 criteria: the number of associated participants, applying a significance level of .05 (corresponding to a factor loading of ±0.34); a minimum of 2 participants per factor; and the Kaiser-Guttman criterion, which suggests retaining factors with eigenvalues of 1.0 or higher [27,39]. For each factor, the rankings of associated participants were combined by calculating weighted averages to create an “idealized Q-sort.” These idealized Q-sorts illustrate how a typical participant with that viewpoint would rank the statements on the grid [27]. Qualitative data from the interviews were incorporated to contextualize and enhance the understanding of the shared viewpoints. No coding or categorization of the qualitative data was conducted prior to the quantitative analysis. After the quantitative analysis revealed the viewpoints, the qualitative data were categorized accordingly. The reporting of the study adhered to the checklist developed by Dieteren et al [40], ensuring transparency and methodological rigor.

### Ethical Considerations

Ethics approval for this study was obtained from the internal review board of Erasmus School of Health Policy & Management, Erasmus University Rotterdam (ETH2324-0759), and all participants provided written informed consent to participate in the study. To support participant understanding, the consent form was written in Dutch at the B1 level and discussed with each patient prior to signing. All data collected were pseudonymized to protect participant privacy and confidentiality. The key linking participant identities to the data was stored separately on a password-protected file, with access restricted to the research team. Participants received a €20 (~23.5 USD) gift card as compensation for their participation.

## Results

### Overview

In total, 21 individuals participated in the study (Table 2). Only 2 of them had experience with home-based rehabilitation technology, specifically with an app, while the others had no prior experience. The analysis identified 3 factors that represent 3 shared viewpoints, collectively accounting for 54% of the variance. Data from 19 of the 21 participants were included, as 2 Q-sorts were confounded. Viewpoint 1 was supported by 9 participants, viewpoint 2 by 7 participants, and viewpoint 3 by 3 participants. The correlations between the viewpoints were moderate, with coefficients ranging from *r*=0.47 to *r*=0.59. The idealized Q-sort for the 3 viewpoints is presented in Table 1.

**Table 2.** Characteristics of participants (N=21).

Characteristics	Values
Sex (female), n (%)	11 (52)
Age (years)	
Mean (SD)	54 (11)
Range	31‐67
Marital status, n (%)	
Married	10 (47)
Living with a partner	4 (19)
Widowed	1 (5)
Single	6 (29)
Education, n (%)	
Secondary education	2 (10)
Vocational education	9 (43)
Higher education	10 (47)
Rehabilitation phase, n (%)	
Undergoing rehabilitation	14 (67)
Finished rehabilitation	7 (33)
Type of acquired brain injury, n (%)	
Stroke	20 (95)
Brain tumor	1 (5)
Type of impairment, n (%)	
Motor only (wheelchair dependent)	7 (33)
Motor only (not wheelchair dependent)	6 (29)
Motor, cognitive and language (wheelchair dependent)	3 (14)
Language only^a^	1 (5)
Cognitive and language only^a^	4 (19)

a Participants who previously experienced motor impairments but have recovered motor function.

### Viewpoint 1: Technology Supporting Fast Recovery

In total, 9 participants held viewpoint 1. The majority were male participants (n=6), and 5 had attained higher education qualifications. Of these, 3 lived alone. Motor impairments requiring the use of a wheelchair were reported by 6 participants, and 3 of them also experienced language and cognitive difficulties. An additional 3 participants had motor impairments but did not require a wheelchair. All participants were engaged in rehabilitation.

Participants in this group believed that a faster recovery (statement 1, +4) and the quicker achievement of rehabilitation goals (statement 2, +4) were the most important factors when deciding to use rehabilitation technology at home. They expressed a strong desire to return to activities they had engaged in before their ABI: “I am a teacher and a sculptor. (…) and I need both of my hands for that. I’m hoping for a quick recovery. The sooner, the better” (Participant 11).

This focus on speed also influenced their preferences for technology that could support their recovery. Participants preferred devices that could be set up quickly, ideally within 5 minutes (statement 13, +2), and that were quick and easy to understand (statement 9, +1). They felt that complex or time-consuming setups could lead to frustration, reduced performance, and diminished motivation. Participant 14 explained:

*If I have to think too much about something, my body doesn’t function as well. It’s either thinking or focusing on my body; I can’t do both at the same time. If the setup of the technology requires too much effort, it takes away from my movement, making it not worth using.*

Additionally, some participants emphasized the importance of simplicity, as they anticipated needing assistance from family members:

*I can’t do much on my own, so anything I can manage by myself is a bonus. Otherwise, someone else has to help me. My girlfriend is usually around, but she’s not very good with this sort of thing, so it’s helpful if the technology is simple, like having just one button.*

[Participant 18]

Participants in this group placed significant value on family support (statement 15, +2), viewing it as essential for both practical help with using rehabilitation technology and emotional reassurance during decision-making. Participant 14 noted: “They influence my choices. It’s reassuring to know that they support my decisions and back me up.” Some participants mentioned that their family members sought additional information through the media to better understand their conditions and explore solutions. Participant 15 stated:

*My family influences my decisions; they are important to me. They try to gain more knowledge through social media, but the information they find is often too limited and doesn’t apply to my situation.*

Participants often regarded media coverage, whether from television (statement 19, −4) or social media (statement 18, −4), as unreliable and irrelevant to their recovery. They believed that recovery is unique to each individual, which also shaped their aversion to comparing progress with peers (statement 24, −1): “Everyone is unique, and every injury [referring to the aftermath of a stroke] is different, so there is no point in making comparisons” (Participant 19). This perspective also influenced their attitude toward game-like exercises (statement 27, −3), which they often associated with competition against peers. They found this unhelpful and preferred practical exercises: “There can be a competitive element, but it shouldn’t be against someone else, as such comparisons are not meaningful” (Participant 21). Participant 14 added: “It’s not something I need. While having some variety can be nice, I’ve gained much more from working hard and focusing on the practical exercises.”

Participants in this group expressed minimal concern about safeguarding their privacy (statement 31, −3), believing their data would be secure: “I think it will be fine” (Participant 11). Additionally, they were open to using technology to replace certain aspects of their rehabilitation center–based treatment (statement 34, −1). This openness was primarily driven by concerns about the difficulties they might face when traveling to the center after transitioning to outpatient care: “I don’t think it’s impossible to come here, but it will be difficult since I’m in a wheelchair” (Participant 11).

### Viewpoint 2: Technology Supporting Independence and Self-Control

In total, 7 participants aligned with this viewpoint, the majority of whom were female (n=4). Of these, 4 lived alone, and 4 had attained higher education qualifications. Previous motor impairments were reported by 4 participants, who now experienced only speech and memory difficulties. Motor impairments were still experienced by 3 participants: 1 required a wheelchair, while 2 did not. Of the 7 participants, 4 had completed rehabilitation, while 3 were still undergoing it.

This group highly valued their autonomy and independence during recovery. They prioritized the flexibility to choose when to exercise (statement 5, +4) and control over their recovery process (statement 3, +4). Their aversion to being told what to do made the ability to decide when to exercise particularly important:

*I really dislike being told, “You have to do this now.” That’s why I don’t go to physical therapy. For example, if I’m supposed to go on Tuesday at ten o’clock, I might not feel up to it or might be too tired to go.*

[Participant 2]

For them, the autonomy to choose when and how to exercise was crucial for maintaining a sense of control:

*It’s about making my own decisions regarding my pace, whether I go fast or slow. Having the freedom to choose what I can do is important.*

[Participant 3]

The emphasis on autonomy and independence also extended to their preferences for technology. They valued the ability to use it independently (statement 12, +3), as requiring assistance diminished their autonomy, reduced flexibility, and created an unwelcome burden for others: “It would be great not to rely on others to use it. This would also make things easier for the people around me” (Participant 8). Those living alone found technology that required assistance particularly inconvenient: “It doesn’t seem very practical to have technology at home that can only be used with others. I live alone” (Participant 4). As participants emphasized the importance of using technology independently, they attached less value to technology with varying levels of difficulty (statement 26, −1): “There should be some adaptability in difficulty (...) but that’s not the most important thing” (Participant 3).

To maintain their independence, participants emphasized the importance of gaining insight into their progress (statement 22, +3). They wanted to monitor their achievements and identify areas for improvement: “Insight into my own progress is important because it helps me understand where I need to improve or what I need to work on” (Participant 13). They also valued direct feedback during exercises (statement 23, +1) to ensure that tasks were performed correctly: “That seems useful to me, because otherwise, you don’t gain anything from it” (Participant 7). Additionally, they stressed the need for technical support, such as a helpdesk (statement 28, +1), to resolve issues independently without relying on family or friends:

*If something doesn’t work and you can’t fix it yourself, it is crucial to have someone available to help immediately. Otherwise, you might have to wait a couple of days until someone has time to help, which feels pointless.*

[Participant 3]

Similar to viewpoint 1, participants in this group placed little emphasis on comparing their progress with peers (statement 24, −2), believing that recovery is unique to each individual: “I don’t think it’s important to compare, because everyone is different” (Participant 2). Their focus was primarily on their own recovery, rather than on that of others: “I don’t really think about others in that regard. For me, it’s about focusing on myself” (Participant 3).

In contrast to viewpoint 1, participants in this group placed less emphasis on achieving a faster recovery (statement 1, +1) or reaching rehabilitation goals quicker (statement 2, 0). They viewed recovery as a gradual process rather than something to rush: “A faster recovery process? No, I don’t think it needs to be any quicker. It [recovery] is important, but it doesn’t have to happen fast” (Participant 4). In addition, being able to set up technology within 5 minutes (statement 13, −1) was not a priority, as they appreciated the learning opportunity provided during the installation: “No, it doesn’t matter if setting it up takes a little longer. While doing it, you’re also learning and gaining a better understanding of how it [the technology] works” (Participant 3). Moreover, participants in this group placed significant emphasis on safeguarding their privacy (statement 31, +2), more so than those in viewpoint 1. Some were concerned about the potential misuse of their health data: “Of course, this is important because these medical details are relevant to insurance companies” (Participant 4). Others expressed a strong desire for confidentiality: “Not everyone needs to know what I have. My information should remain private” (Participant 3).

### Viewpoint 3: Technology as a Supporting Partner

This viewpoint was held by 3 female participants, all of whom lived with others. Their educational backgrounds varied, including secondary education, vocational education, and higher education qualifications. Among them, 1 participant experienced language-related difficulties, while the other 2 experienced motor impairments requiring the use of a wheelchair. Of the 3 participants, 1 had completed rehabilitation, while the other 2 were still undergoing it.

Participants in this group emphasized the importance of receiving external insights to guide their recovery process. They valued gaining insight into their progress through technology (statement 22, +4), particularly because they found it difficult to recognize improvements on their own: “Sometimes, it’s hard to see your progress on your own, so it would be helpful to have that insight” (Participant 12). This desire for insight was complemented by their preference for therapists to actively monitor their recovery (statement 21, +4). Participants highlighted the crucial role of therapists, as they could provide valuable guidance and intervene when necessary. Participant 20 explained: “This is important because therapists can intervene when needed.” Participant 12 elaborated on how therapists could help set the appropriate difficulty level and ensure the recovery process stays on track:

*For example, a therapist might recommend trying a higher level. While you can decide for yourself, they can also offer suggestions and ideas on what else to try. Having someone to guide you can be very helpful.*

The need for external guidance extended beyond professional support, as participants valued insights and encouragement from peers. They stressed the importance of maintaining peer contact (statement 20, 0) as well as receiving recommendations from peers (statement 17, +1). Participants saw peers as a source of practical advice and emotional support. Participant 16 shared: “You can learn from them; they have been through it too.” Participant 12 expanded:

*You can learn from one another. You might get tips, or someone might have figured something out that makes you think, “Oh, that’s a good idea; I’ll try that too.” And while family and friends can offer support, connecting with fellow patients is also valuable, as they truly understand what you’ve been through.*

Peer contact also offered reassurance and positive feedback. Participant 12 recounted:

*I just attended a meeting with people who have recovered well and can really express themselves. We were talking, and I said, “Wow, you speak so well!” They said the same to me, you know. It was really nice to hear.*

This emphasis on positive feedback fostered participants’ interest in comparing their progress with others (statement 24, +1) and receiving rewards for their achievements (statement 25, 0): “It’s nice to see and hear that you’re doing well” (Participant 12).

Similar to participants with viewpoint 1, but unlike those with viewpoint 2, participants with viewpoint 3 emphasized the importance of a faster recovery (statement 1, +3) and the quicker achievement of rehabilitation goals (statement 2, +3). Participant 16 expressed: “Well, isn’t that what we all want?” However, they also recognized the need for patience, as participant 20 explained: “I want to recover as quickly as possible, but the body also needs time to adjust.” This nuance suggests that, while speed was a priority, it was not the most important factor influencing their decision to use technology at home. Participants with viewpoints 1 and 3 also shared a similar outlook on privacy, expressing minimal concern about safeguarding their personal information (statement 31, −4). They trusted that privacy measures were sufficient and felt that they had little to hide. Participant 20 commented: “I think that it will be fine. I also don’t have much to hide*.*” For those with viewpoint 3, this confidence extended to safety (statement 30, 0), as they believed the technology posed minimal risks.

In contrast to viewpoint 1, but aligning with viewpoint 2, participants with viewpoint 3 did not consider the ability to set up technology within 5 minutes (statement 13, −2) a priority. They expressed a relaxed attitude toward setup time, prioritizing functionality over speed. Participant 20 explained: “Five minutes? Why? There’s plenty of time in a day.” Participant 12 added, “Fifteen minutes would be fine with me as well. I want the technology to work properly.”

Participants with viewpoint 3 placed less importance on the convenience of recovery at home (statement 4, 0), cost reimbursement (statement 29, −2), and the size of the technology (statement 32, −3) compared to those with viewpoints 1 and 2. They recognized that recovering at home could be convenient but argued that its relevance varies depending on the length of the rehabilitation period. They also stressed that being in the rehabilitation center is important for their recovery. In addition, they believed that the technology should be reasonably priced and of a manageable size. Participant 12 explained: “I do not consider costs when it comes to rehabilitation. That is not the priority; regaining my life is. However, it should still be affordable.”

### The Consensus Among the 3 Viewpoints

Despite differences among the 3 viewpoints, participants agreed on several factors that are important to their decision to use rehabilitation technology at home. One point of consensus was the potential to reduce dependence on others (statement 7, +1, +1, +2). Many participants expressed frustration with relying on family members for everyday tasks and viewed rehabilitation technology as a way to regain autonomy. Participant 8 explained:

*I really value my independence. For example, last weekend (...), I couldn’t get a drink. I had to wait for my partner to bring it to me, which I don’t want to keep doing. If the technology helps me practice these tasks, it would be great as it allows me to become less dependent.*

In addition, participants consistently emphasized the importance of certain features in rehabilitation technology. One key feature was direct feedback during exercises (statement 23, +3, +1, +3), which they believed was necessary for effective rehabilitation. Participant 11 noted: “Otherwise, you don’t know if you’re doing things correctly or incorrectly. I think that’s really important in any learning process.” Other important features emphasized by the participants included simplicity (statement 8, 0, 0, +1) and technology that could be understood quickly (statement 9, +1, 0, −1). Participants noted that difficulties in these areas might lead to frustration and reduce engagement. However, they did not consider being able to understand the technology without any explanation (statement 11, 0, −1, −2) to be essential. Some felt they could learn on their own, while others were comfortable receiving help or explanations from a therapist or family member.

Participants also agreed on several factors related to social influences. Approval from therapists (statement 14, +1, +2, +2) was considered very important, as therapists were seen as trusted advisers in decisions about home-based rehabilitation technology. In contrast, information from social media (statement 18, −4, −4, −4) and television (statement 19, −4, −4, −2) was deemed unimportant to their decision-making, either because participants used these platforms infrequently, questioned their reliability, or found them irrelevant. Likewise, support from friends (statement 16, −2, −2, −3) was not regarded as an important factor in deciding whether to use home-based rehabilitation technology.

Finally, participants agreed that reducing travel to the rehabilitation center (statement 6, −2, −2, −1) was not a major factor in their decision to use rehabilitation technology. Many lived close to the center, making travel a nonissue: “No, I live nearby, so it doesn’t matter to me. I can even walk to the centre” (Participant 4). For those who lived farther away, tailored transport options, which are partly reimbursed by health insurers under certain conditions, minimized the challenge. Participant 2 shared: “I have tailored transport, so travelling to the centre isn’t really an issue; it’s all arranged for me.”

## Discussion

### Principal Findings

This study identified three viewpoints on the adoption of rehabilitation technology for home use by rehabilitation patients with ABI: (1) technology supporting fast recovery, which prioritizes quick outcomes and ease of use; (2) technology supporting independence and self-control, which emphasizes autonomy and personal control; and (3) technology as a supporting partner, in which guidance and validation are highly valued. These findings highlight the diverse viewpoints of rehabilitation patients with ABI, suggesting that a tailored approach may be more effective than a one-size-fits-all approach.

### Technology Supporting Fast Recovery

Participants with this viewpoint were predominantly male, cohabiting, and more severely impaired compared to those in other viewpoints. All were actively undergoing rehabilitation. They emphasized the importance of technology in achieving rapid recovery and meeting rehabilitation goals. This viewpoint seems to align with literature suggesting that male participants often prioritize results [15,23] and that individuals in the early stages of rehabilitation focus on the practical aspects of recovery [41]. Additionally, participants preferred devices that were easy to use and quick to set up, ideally within 5 minutes, to minimize frustration. This finding aligns with those of Lin et al [42] and Klaic and Galea [19], who noted that tasks with high cognitive load can lead to increased frustration and reduced performance. Features enabling progress comparisons or competitive, game-like formats were of little interest to participants in this group. This observation contrasts with previous studies [20,30,36]. However, those studies focused on individuals in the chronic phase of recovery, whereas participants in this group were actively undergoing rehabilitation and prioritized personal recovery and practical exercises. Moreover, participants emphasized the uniqueness of each recovery journey, which reduced the relevance of comparative metrics. This seems to align with Festinger’s social comparison theory [43], which posits that individuals are less likely to compare themselves with others when there are substantial differences. Rehabilitation technology for this group should therefore focus on tracking individual milestones rather than comparative metrics. Family appeared to play an important role in decisions about adopting home-based rehabilitation technology. Participants noted that practical assistance (eg, help with setting up devices) and emotional support (eg, approval from family members) influenced their decision to adopt rehabilitation technology. This finding aligns with Schladen et al [44] and Nasr et al [36], who suggested that family involvement often plays a key role in decision-making. For this group, it is important that rehabilitation technology is designed to accommodate the needs of both users and their families and that family members are involved in the decision-making process when patients are presented with the option to use such technology.

### Technology Supporting Independence and Self-Control

This viewpoint was predominantly expressed by female participants, most of whom were living alone. While most no longer experienced motor impairments and had completed rehabilitation, they continued to report cognitive and language difficulties. Autonomy and independence were viewed as important aspects of rehabilitation technology. This viewpoint seems to align with literature suggesting that female participants often adopt a process-oriented approach [15,23]. Additionally, the emphasis on autonomy and independence, as noted in previous studies [31,32,36], suggests that reliance on caregivers or professional support may act as a barrier to adopting rehabilitation technology in this group. Participants also highlighted the importance of gaining insight into their progress and receiving feedback during exercises. While feedback can motivate users [32,33,36], participants in this group primarily viewed it as a tool for improving performance. Participants further emphasized the importance of features such as access to a helpdesk and robust privacy safeguards, which is consistent with findings by Brouns et al [34]. These aspects should be prioritized in the design of rehabilitation technology for this group. Like the first viewpoint, participants placed limited value on comparing their progress with others. Instead, they emphasized the uniqueness of their recovery journeys. As a result, technology developed for this group may be most effective if it focuses on tracking individual progress and enables users to monitor it independently.

### Technology as a Supporting Partner

This viewpoint was exclusively expressed by cohabiting female participants, who reported a range of impairments, with the majority still undergoing rehabilitation. Guidance and validation from external sources were considered important in their decision to adopt the technology. This viewpoint seems to align with existing literature, suggesting that female participants are more likely to adopt a process-oriented approach and tend to place greater emphasis on external opinions [15,23]. Furthermore, participants in this group emphasized the importance of tracking their own progress with technology, particularly as improvements are not always visible, a challenge also noted by Lavis et al [45]. Technology with clear progress-tracking features, such as charts or milestones, could help address this need. Additionally, therapist involvement was highlighted as crucial for the adoption of home-based rehabilitation technology, as participants noted the importance of receiving feedback, guidance, and help with adjusting difficulty levels. This aligns with prior research that underscores the value of therapist support in rehabilitation [19,30,33]. Moreover, participants valued staying connected with peers, receiving recommendations, comparing progress, and earning rewards. These preferences align with previous studies [34,35,45] and suggest that peer-focused communication features, comparative metrics, and reward systems could be beneficial for this group. Similar to the first viewpoint, participants in this group shared a desire for quicker recovery and goal achievement. However, they also acknowledged the importance of patience throughout the process. This indicates that, although fast recovery was valued, it was not the sole consideration in this group’s decision to adopt home-based rehabilitation technology. Such reflections are consistent with prior studies [19,30,32,33]. Finally, although cost and size were noted as considerations [20,31,33,34], participants in this group expressed a willingness to invest in technology they perceived as helpful for their recovery.

### The Consensus Among the Viewpoints

Participants agreed on several factors as important to their decision to adopt technology: reducing reliance on others, receiving immediate feedback, ensuring ease of use, and obtaining therapist approval. The emphasis on independence aligns with previous studies, which have identified it as a key rehabilitation outcome from the patient’s perspective [46]. Immediate feedback during exercises was also considered important, consistent with earlier findings [20,32,36]. However, as this study did not explore the optimal format for delivering feedback, further investigation is warranted. Ease of use was also frequently cited as important [10,15,19,30-33,35], suggesting that ideal technology should be intuitive. However, participants in this study noted that the technology does not need to be entirely self-explanatory, as long as support is available, whether through written instructions, professionals, or family members. Moreover, therapist approval emerged as particularly important in the decision to adopt technology, reflecting previous research findings [36]. In contrast, participants showed disinterest in mainstream media (eg, social media and television) when deciding whether to adopt rehabilitation technology. This may be attributed to the generally older age of participants [47] or to the belief that each recovery journey is unique. Similarly, support from friends was mostly considered less important in the decision to adopt. Notably, avoiding travel to rehabilitation centers was not a priority for most participants. This differs from other studies [20,31,33,34], where it was seen as a major advantage. This contrast may be due to the fact that participants in this study lived nearby and that transportation costs are partially reimbursed by Dutch health insurers [48].

### Implications

The findings of this study highlight the importance of a personalized, user-centered approach to the development and implementation of home-based rehabilitation technologies. Rehabilitation patients with ABI hold diverse viewpoints regarding the adoption of such technologies, underscoring the need for flexible and adaptable design. Actively involving patients in the development process can help ensure that these tools align with their expectations and support sustained engagement [10,11].

In terms of design, the findings suggest several practical implications. Across all viewpoints, participants emphasized the importance of intuitive interfaces and immediate feedback. However, the technology does not need to be fully self-explanatory, provided that support is available. Participants in viewpoint 1 seemed to prefer designs that prioritize simplicity and quick setup. Interfaces should therefore offer clear navigation and minimize the number of steps required. Social or peer-related features were considered less important and may be regarded as nonessential for participants with this viewpoint. For viewpoint 2, participants seemed to value designs that support independent use. This includes enabling self-paced interaction, personal goal setting, and individual progress tracking. As with viewpoint 1, social or peer-related features were not prioritized and may be considered nonessential. Participants in viewpoint 3 seemed to prefer designs that incorporate external guidance and validation. Features enabling therapist communication and peer support may therefore be particularly valuable for participants with this viewpoint. Additionally, features such as motivational feedback and rewards appear to be especially appropriate.

In terms of implementation, the findings also offer practical guidance. Motivations for using rehabilitation technologies varied among participants. While some focused on functional recovery, others prioritized maintaining autonomy or receiving consistent support. This variation highlights the need for tailored communication strategies that reflect individual goals and expectations. Moreover, therapists’ approval played an important role in rehabilitation patients’ decisions regarding adoption. As shown in this study and in the work of Nasr et al [36], patients often rely on therapists’ guidance when deciding whether to use such tools. To guide rehabilitation patients effectively, therapists may benefit from training programs that build confidence and skills to recommend and evaluate these technologies.

### Strengths and Limitations

A key strength of this study lies in the use of Q-methodology, which offers a structured yet flexible approach to exploring and comparing subjective viewpoints. This method is particularly well suited for including vulnerable populations, such as individuals with language or cognitive impairments [27,49-51], as it allows statements to be tailored to the target group and accommodates nonverbal communication. In this study, it enabled us to include rehabilitation patients with such impairments and to explore their viewpoints in depth. Given its strength in engaging vulnerable populations in research [49-51], we argue that Q-methodology could be used more frequently in rehabilitation research to help identify and understand the needs of vulnerable patient groups and to support more person-centered research.

However, several limitations should be considered when interpreting our findings. First, our analysis drew on the UTAUT framework [15] to interpret participants’ views on technology adoption. While alternative behavioral models such as self-determination theory [52] or the health belief model [53] could have offered additional conceptual perspectives, we chose not to integrate them, as key constructs (eg, competence and perceived benefits) were already reflected in the data. This choice to focus only on the UTAUT framework may have limited the breadth—but not the depth—of the interpretation presented. Second, the study was conducted at a single rehabilitation center located in an urban area. As patient demographics and health care practices can vary across settings, future research should involve multiple centers, particularly in rural or less densely populated areas, to enhance the generalizability of findings. Third, the sample showed limited ethnic diversity, with only 1 participant each from Surinamese, German, and Croatian backgrounds. This may restrict the applicability of our findings to more ethnically diverse populations. Moreover, the majority of participants had experienced strokes, which may limit the relevance of the findings to individuals with other forms of ABI. Future studies should aim to include a broader range of injury types and participants from more diverse backgrounds to validate and expand upon our results. Finally, individuals with visual impairments were excluded from the study. Although it is possible to read the statements aloud, the second part of the method requires participants to physically sort a full set of written statements on a visual grid. This task relies heavily on visual-spatial processing. During piloting, participants with visual impairments experienced confusion and frustration. To prevent further distress and ensure methodological consistency, we decided to exclude individuals with significant visual impairments. This exclusion may have affected the representativeness of the sample, as their perspectives on technology adoption may differ in meaningful ways.

### Conclusions

This study identified three viewpoints regarding the factors that rehabilitation patients with ABI consider important for the adoption of home-based rehabilitation technology: (1) technology supporting fast recovery, (2) technology supporting independence and self-control, and (3) technology as a supporting partner. These findings demonstrate that rehabilitation patients with ABI differ in both the design features they prioritize and the motivations that drive their use. As such, a one-size-fits-all approach to the development and implementation of home-based rehabilitation technology is unlikely to be effective.

## List of abbreviations

ABI: acquired brain injury
Q-study: Q-methodology study
UTAUT: unified theory of acceptance and use of technology
